# An efficient *i*-GONAD method for creating and maintaining lethal mutant mice using an inversion balancer identified from the C3H/HeJJcl strain

**DOI:** 10.1093/g3journal/jkab194

**Published:** 2021-06-03

**Authors:** Satoru Iwata, Takahisa Sasaki, Miki Nagahara, Takashi Iwamoto

**Affiliations:** 1 Center for Education in Laboratory Animal Research, Chubu University, Kasugai, Aichi 487-8501, Japan; 2 Department of Biomedical Sciences, College of Life and Health Sciences, Chubu University, Kasugai, Aichi 487-8501, Japan; 3 College of Bioscience and Biotechnology, Chubu University, Kasugai, Aichi 487-8501, Japan

**Keywords:** *in vivo* electroporation, *i*-GONAD, CRISPR/Cas9, inversion balancer, lethal mutation

## Abstract

As the efficiency of the clustered regularly interspaced short palindromic repeats/Cas system is extremely high, creation and maintenance of homozygous lethal mutants are often difficult. Here, we present an efficient *in vivo* electroporation method called improved genome editing via oviductal nucleic acid delivery (*i*-GONAD), wherein one of two alleles in the lethal gene was selectively edited in the presence of a non-targeted B6.C3H-*In(6)1J* inversion identified from the C3H/HeJJcl strain. This method did not require isolation, culture, transfer, or other *in vitro* handling of mouse embryos. The edited lethal genes were stably maintained in heterozygotes, as recombination is strongly suppressed within this inversion interval. Using this strategy, we successfully generated the first *Tprkb* null knockout strain with an embryonic lethal mutation and showed that B6.C3H-*In(6)1J* can efficiently suppress recombination. As B6.C3H-*In(6)1J* was tagged with a gene encoding the visible coat color marker, *Mitf*, the *Tprkb* mutation could be visually recognized. We listed the stock balancer strains currently available as public bioresources to create these lethal gene knockouts. This method will allow for more efficient experiments for further analysis of lethal mutants.

## Introduction

Clustered regularly interspaced short palindromic repeats (CRISPR)/Cas9-mediated mutagenesis has been widely used to disrupt genes in mice, rats, zebrafish, fruit flies, and nematodes (Pennisi 2013). However, lethal genes are often difficult to disrupt because both alleles are frequently disrupted simultaneously (Gurumurthy *et al.* 2019a). Approximately one-third of mouse genes are essential for life, and the mouse null-phenotypes for 61–62% of genes are currently unknown (Hrabe de Angelis *et al.* 2015; Dickinson *et al.* 2016). For efficient knockout studies of disease-causing and essential genes, it is crucial to establish methods that aid in the generation and analysis of lethal mutants.

A recent study reported that microinjection of the CRISPR/Cas9 system into one blastomere of two-cell embryos can be performed to efficiently generate mouse strains carrying heritable lethal mutations (Wu *et al.* 2019). However, this method requires specialized equipment and highly skilled personnel. To overcome this limitation, we recently introduced a recessive lethal knockout by targeting an allele in F1 hybrid mice via improved genome editing via oviductal nucleic acids delivery (*i*-GONAD) of Cas9 and guide RNAs (gRNAs) into mouse zygotes (Iwata *et al.* 2019). The *i*-GONAD method reported by Ohtsuka *et al.* (2018) employs intraoviductal instillation of genome editing components and subsequent electroporation of the oviduct and, therefore, does not require handling of preimplantation embryos. A recent study showed that compared with microinjection, electroporation results in a higher rate of embryo survival and development (Alghadban *et al.* 2020). However, statistical calculations indicated that even speed congenic approaches require a minimum of four backcrosses to eliminate a fully unwanted donor genome from F1 hybrid mice (Rogner and Avner 2003). Moreover, maintenance of deleterious mutations in heterozygotes from one generation to the next requires the selection of heterozygous individuals, which is labor-intensive.

In this study, we developed the *i*-GONAD method in which one allele was selectively edited using a B6.C3H-*In(6)1J* inversion identified from C3H/HeJJcl. Appropriately marked inversions were used as balancer chromosomes to maintain mutations in the corresponding chromosomal region (Zheng *et al.* 1999; Nishijima *et al.* 2003). Using this method, we generated the first *Tprkb* null knockout mouse with an embryonic lethal mutation that was stably maintained in heterozygotes. Finally, we listed the inversion balancer strains currently available via public bioresources to create these lethal gene knockouts using the above method.

## Materials and methods

### Animal strains

C57BL/6NCrSlc, C3H/HeJYokSlc (Japan SLC, Shizuoka, Japan), and C3H/HeJJcl mice (CLEA Japan, Tokyo, Japan) were used in this study. The animals were maintained at a constant temperature (22  ± 2°C) and humidity (50 ± 10%), with a 12-hours light/12-hours dark cycle. All animal experiments were approved by the Institutional Animal Care and Use Committee of Chubu University (Permit Numbers #2910066, #2910067 at Chubu University) and were conducted in accordance with institutional guidelines.

### Whole-genome sequencing analysis

Raw sequencing reads of C3H/HeJ were previously sequenced by Keane *et al.* (2011) and deposited in the DNA Data Bank of Japan (DDBJ) Sequence Read Archive (DRA) (https://ddbj.nig.ac.jp/DRASearch/, Accession: ERR008069 and ERR008070). Sequence read mapping was performed using BWA-mem software implemented in the MASER pipeline (Kinjo *et al.* 2018). Inversion *In(6)1J* breakpoints were identified as reported previously (Chen *et al.* 2009; Fan *et al.* 2014). Regions in which BreakDancer identified large inversion polymorphisms and breakpoints were visually validated using Integrative Genomics Viewer (Thorvaldsdóttir *et al.* 2013). Polymerase chain reaction (PCR) was performed with Ex Taq polymerase, and Sanger sequencing confirmed each breakpoint. PCR primers used to validate the inversion breakpoints are listed in Supplementary Table S1.

### Test for recombination suppression

To examine whether recombination was suppressed in *In(6)1J*, homozygous *In(6)1J* (C3H/HeJJcl background) females were mated with C57BL/6NCrSlc males, and the F1 heterozygotes were further backcrossed for six generations to obtain C57BL/6NCrSlc mice. The single-nucleotide polymorphism (SNP) genotype of each region was determined by PCR-restriction fragment length polymorphism (RFLP) analysis. We identified four SNPs (described in dbSNP) with mismatch PCR-RFLP based on the Mouse Genomes Project at Wellcome Sanger Institute (https://www.sanger.ac.uk/sanger/Mouse_SnpViewer/rel-1505). PCR primers were used to amplify a genomic sequence containing a restriction site in C3H/HeJ mice but not in C57BL/6N mice. Following PCR amplification, the PCR products were digested for 4 hours at 37°C with 5 units of restriction enzyme and then analyzed by 1.2% agarose gel electrophoresis. The PCR primers used to validate recombination suppression are listed in Supplementary Table S1.

### CRISPR solutions

Allele-specific CRISPR guide RNAs were designed using an SNP data retrieval utility, such as https://phenome.jax.org/snp/retrievals, and cleavage efficiencies were retrieved from CHOPCHOP (Labun *et al.* 2019, http://chopchop.cbu.uib.no/) (Supplementary Table S2). CRISPR RNP consists of Alt-R S.p. Cas9 Nuclease 3NLS (Integrated DNA Technologies, Coralville, IA, USA) and a custom guide RNA (crRNA): tracrRNA duplex, which includes the crRNA and a universal structural RNA (tracrRNA) (Integrated DNA Technologies). crRNA and tracrRNA were heated to 95°C for 10 minutes and slowly cooled to 25°C. This crRNA: tracrRNA duplex and the Alt-R S.p. Cas9 Nuclease 3NLS were incubated at 25°C for 10 minutes to form the RNP complex.

### *i*-GONAD method

To synchronize the estrous cycle of female mice, 8–12-weeks-old female mice were injected intraperitoneally with 2.4 IU pregnant mare serum gonadotropin and mated with 8–24-weeks-old males 48 hours later, as previously described (Kobayashi *et al.* 2020). The presence of copulation plugs was confirmed the next morning via visual inspection, and plug-positive mice were subjected to *i*-GONAD experiments, as previously described (Ohtsuka *et al.* 2018; Gurumurthy *et al.* 2019b). To generate a lethal gene deletion, the following concentrations of CRISPR solutions were used: 540 ng/μl Alt-R S.p. Cas9 Nuclease 3NLS, 33 μM upstream and downstream crRNA/tracrRNA, and 0.05% Fast Green FCF (Wako, Osaka, Japan) marker diluted in Opti-MEM (Thermo Fisher Scientific, Waltham, MA, USA). Prior to electroporation, females were anesthetized with a mixture of medetomidine (0.75 mg/kg), midazolam (4 mg/kg), and butorphanol (5 mg/kg). The CRISPR mixture (1 μl) was injected into the oviductal lumen upstream of the ampulla with a glass micropipette, which was made using a vertical capillary puller (NARISHIGE, Tokyo, Japan). Following injection of CRISPR solutions, the oviduct regions were grasped using tweezer electrodes (CUY652P2.5 × 4; Nepa Gene, Chiba, Japan), and electroporation was performed as previously described (Kobayashi *et al.* 2020) using a NEPA21 (Nepa Gene). The following parameters were used for electroporation: poring pulse (voltage: 40 V; pulse length: 5.0 ms; pulse interval: 50 ms; number of pulses: 3; decay rate: 10%; polarity: ±), transfer pulse (voltage: 10 V; pulse length: 50 ms; pulse interval: 50 ms; number of pulses: 3; decay rate: 40%; polarity: ±). Following electroporation, we placed the oviducts back in their original location and sutured the incisions. Following the operation, atipamezole hydrochloride (0.75 mg/kg) was intraperitoneally injected to reverse the effects of medetomidine.

### Analysis of CRISPR/Cas9-engineered mice

To screen for CRISPR/Cas9-induced deletions, genomic DNA was isolated from the tails or ears of founder mice using lysis buffer [100 mM NaCl, 200 mM sucrose, 10 mM ethylenediaminetetraacetic acid, 300 mM Tris (pH 8.0), and 1% sodium dodecyl sulfate], and DNA was examined by PCR amplification. PCR products were cloned into the pTAC-1 vector (Biodynamics, Tokyo, Japan), and the sequences of individual clones were determined by Sanger sequencing (Eurofins Genomics, Tokyo, Japan). The PCR primers used for genotyping are listed in Supplementary Table S1.

### RT-PCR

Reverse transcriptase (RT)-PCR was performed using total RNA. Total RNA was isolated from ear tissue using ISOSPIN Cell & Tissue RNA (Nippon Gene, Tokyo, Japan). Template cDNA was obtained using ReverTra Ace qPCR RT Master Mix (Toyobo, Osaka, Japan). The RT-PCR products were directly analyzed by Sanger sequencing (Eurofins Genomics, Tokyo, Japan). The primers used for RT-PCR are listed in Supplementary Table S1.

### Test for the balancer chromosome

B6.C3H-*In(6)1J* was selected to examine whether it could balance a recessive lethal mutation. A homozygous B6.C3H-*In(6)1J Mitf^em1Cu^* male was mated with a heterozygous *Tprkb^em1Cu^* female. Following inbreeding of F1 *Tprkb^em1Cu^*/B6.C3H-*In(6)1J Mitf^em1Cu^* mice, their offspring were phenotypically distinguishable. A schematic diagram of the assay and the expected results is depicted in Figure 5, A and B. In the assay, two PCR reactions are used to genotype the *Tprkb* deletion. The first reaction utilizes the external primers to specifically amplify only the *Tprkb*-deleted product. In the second reaction, the internal primers are used to detect the *Tprkb*-exon 10 fragment, which was engineered to be deleted by the genome editing. Crude DNA derived from each embryo was prepared as a PCR template according to published methods (Shea and Geijsen 2007; Martin and Cockroft 2008). The *Tprkb^em1Cu^* genotype of each individual was determined by PCR with the primers listed in Supplementary Table S1.

### Statistical analysis

For Mendelian genotype ratios of progeny obtained from sibling mating between *Tprkb^em1Cu^/In(6)1J* mice, the chi-square test was performed using Excel version 16.36 (Microsoft, Redmond, WA, USA). The threshold for statistical significance was *P* < 0.05.

### Data availability

The B6.C3H-*In(6)1J Mitf^em1Cu^* mice used in this study are available at the RIKEN BioResource Research Center and Center for Animal Resources. The C3H/HeJ genome assembly is available for download from the DRA search of DDBJ (https://ddbj.nig.ac.jp/DRASearch/). Supplementary material is available at figshare: https://doi.org/10.25387/g3.14677524.

## Results and discussion

### Isolation of inversion B6.C3H-*In(6)1J* on chromosome 6 from C3H/HeJJcl

**Figure 1:**
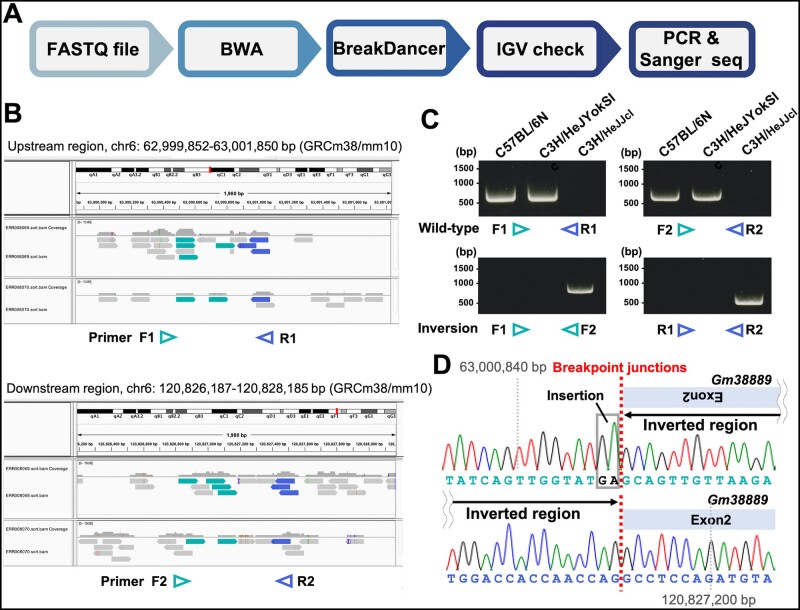
Verification of *In(6)1J* breakpoints from the C3H/HeJ genome by whole-genome sequencing. (A) Flowchart of *In(6)1J* breakpoint analysis using whole-genome sequencing data. (B) IGV browser of C3H/HeJ data (Accession: ERR008069 and ERR008070) aligned to the mouse genome (GRCm38/mm10). Green and blue reads indicate that they are mapped to the reverse strand. Normal reads are shown in gray. Primers (arrows) on chromosome 6 were designed based on the predicted breakpoint. (C) PCR amplification of predicted breakpoint junctions in C57BL/6N, C3H/HeJYokSlc, and C3H/HeJ strains. (D) Sanger sequences corresponding to inversion breakpoint junctions in C3H/HeJJcl. IGV, integrative genome viewer.

Previous studies showed that the inbred mouse strain C3H/HeJ carries an inversion *In(6)1J* on chromosome 6 (Akeson *et al.* 2006; Ackert-Bicknell *et al.* 2007); however, the precise location of the breakpoints remains unknown. To identify *In(6)1J* breakpoints in C3H/HeJ, we retrieved available C3H/HeJ WGS data from the DDBJ DRA (https://ddbj.nig.ac.jp/DRASearch/, Accession: ERR008069 and ERR008070) and analyzed them. The *In(6)1J* breakpoints were predicted from alignment data using the BreakDancer tool version 1.4.5 (Chen *et al.* 2009; Fan *et al.* 2014). We then used Integrative Genomics Viewer (Thorvaldsdóttir *et al.* 2013) to predict candidate breakpoints. We attempted to detect two C3H/HeJ strains (C3H/HeJJcl and C3H/HeJYokSlc) via PCR and Sanger sequencing (Figure 1). In the C3H/HeJJcl strain, one breakpoint was in the intergenic region, whereas the other was in exon 2 of the uncharacterized gene *Gm38889* (Figure 1D). *In(6)1J* encompassed approximately 40% of the chromosome from 63 to 120.8 Mb, which was notably larger than the inversion predicted in previous studies (Akeson *et al.* 2006; Ackert-Bicknell *et al.* 2007). Notably, the strain C3H/HeJYokSlc maintained at Japan SLC, Inc. did not carry *In(6)1J* (Figure 1C); these mice originated from mice distributed by the National Institute of Infectious Diseases in 1985. *In(6)1J* is expected to have occurred in the C3H/HeJ strain after the early 1970s (Akeson *et al.* 2006), suggesting that C3H/HeJYokSlc was derived from the C3H/HeJ strain before 1970.

**Figure 2:**
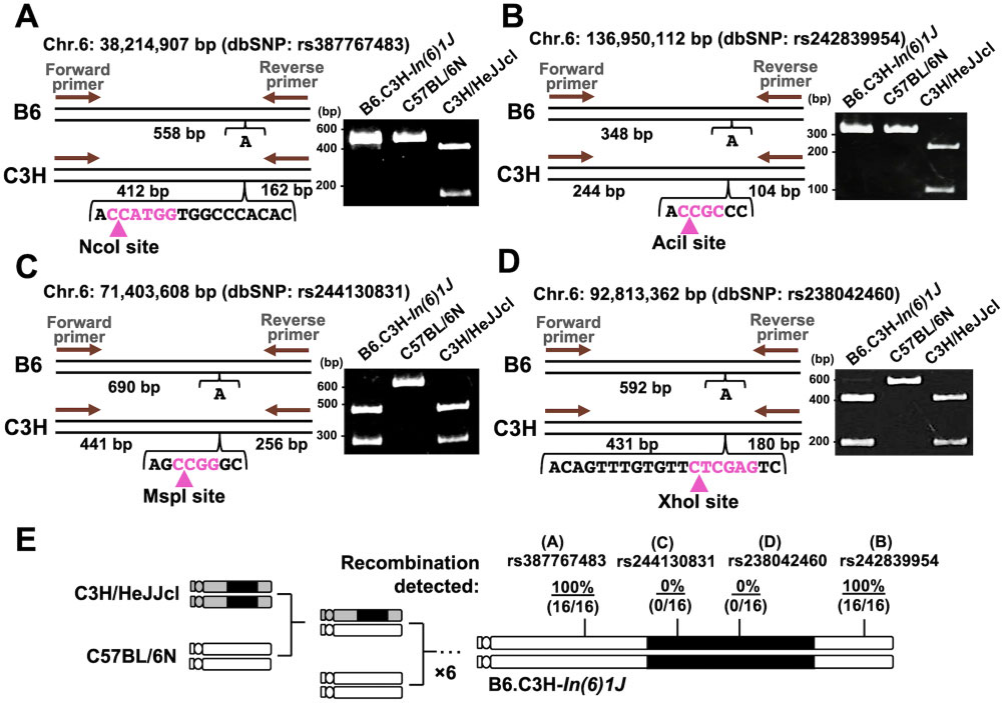
Chromosomal inversion *In(6)1J* can suppress recombination. (A–D) PCR-RFLP assays for recombination frequency determination. The C3H fragment contains a restriction site; however, the B6 fragment does not. (E) B6.C3H*-In(6)1J* congenic strain was produced using six generations of selective backcrossing. Recombination frequencies in the *In(6)1J* inversion. The inversion is indicated by a filled bar. PCR-RFLP, PCR-restriction fragment length polymorphism; B6, C57BL/6N; C3H, C3H/HeJ.

Following identification of the exact position of each inversion breakpoint, the B6.C3H-*In(6)1J* congenic strain was constructed by six generations of selective backcrossing into the C3H/HeJJcl to C57BL/6N background. Previous studies demonstrated that recombination between the wild-type and chromosomal balancer lines does not occur within these inversion events (Zheng *et al.* 1999; Nishijima *et al.* 2003; Iwata *et al.* 2019). To examine whether B6.C3H-*In(6)1J* suppresses crossing over in the inversion interval, we determined the recombination frequencies on chromosome 6. We analyzed four PCR-RFLPs that lie external (dbSNP no. rs387767483 and dbSNP no. rs242839954) and internal (dbSNP no. rs244130831 and dbSNP no. rs238042460) to inversion *In(6)1J*. Among all 16 meioses examined, the external region was recombined with the C57BL/6N strain (Figure 2, A, B, and E). In contrast, there was no crossover event within the inversion, indicating successful recombination suppression (Figure 2, C, D, and E).

### Generation of visible inversion strains using the *i*-GONAD method

**Figure 3:**
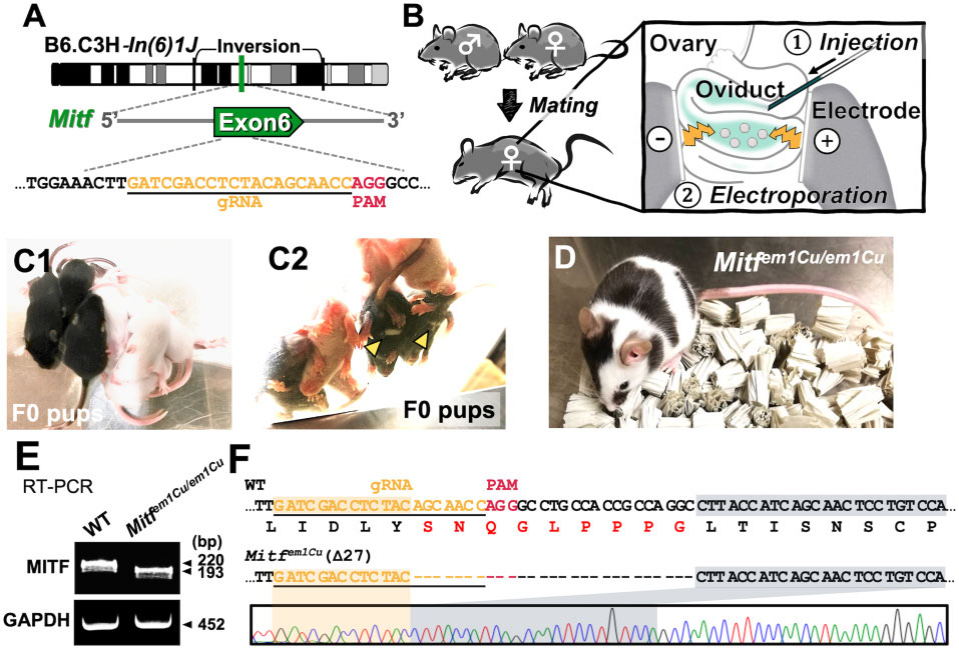
Experimental method to generate visible inversion strains via the *i*-GONAD method. (A) Schematic representation of the gRNA targeting *Mitf* within the *In(6)1J*. The gRNA sequence is underlined in black. The PAM sequence is indicated in red. (B) Experimental procedures for the *i*-GONAD method. The oviducts of a pregnant female were electroporated on day 0.7 of pregnancy. (C1) Dorsal and (C2) ventral views of F0 pups. The white spots on the F0 pups are indicated by yellow arrowheads. (D) Coat color phenotype of inversion mice: *Mitf^em1Cu/em1Cu^* with white spots. (E) RT-PCR analysis of *Mitf* expression in WT and *Mitf^em1Cu/em1Cu^* mice. GAPDH was used as a control. (F) Alignment of sequences corresponding to the *Mitf* cDNA. The *Mitf^em1Cu^* mutation caused an in-frame deletion of 9 amino acids (SNQGLPPPG). gRNA, guide RNA; PAM, protospacer-adjacent motif; GAPDH, glyceraldehyde-3-phosphate dehydrogenase.

To facilitate B6.C3H-*In(6)1J* usage, we induced mutagenesis in the gene for which loss of function was expected to cause a visible phenotype. *Mitf* is a recessive gene within the *In(6)1J* region. *Mitf* mutations show a reduction or lack of pigmentation in the coat, eye, and inner ear of the mouse (Steingrímsson *et al.* 2003). Thus, we performed the *i*-GONAD method to generate a line of B6.C3H-*In(6)1J* mice lacking *Mitf* (Figure 3A). This method can bypass the following three steps: (1) zygote isolation, (2) microinjection, and (3) zygote transfer (Takahashi *et al.* 2015; Ohtsuka *et al.* 2018; Gurumurthy *et al.* 2019b). We injected CRISPR/Cas9 RNPs into the oviduct lumen of a pregnant B6.C3H-*In(6)1J* female and electroporated the oviduct *in vivo* (Figure 3B). Four founder F0 pups were white throughout their bodies, two had belly spot patterns, and one pup was black (Figure 3, C1 and C2). To determine the effect of disruption of *Mitf* on fertility, these mutant mice were mated to C57BL/6N mice and monitored for pregnancy. Breeding experiments revealed that the F0 mice with white spots are fertile; however, the unpigmented mice are sterile. Following backcrossing with C57BL/6N, the B6.C3H-*In(6)1J Mitf^em1Cu^* strain was generated, which eliminated the mosaicism. Heterozygotes for B6.C3H-*In(6)1J Mitf^em1Cu^* had normal coat pigmentation. In contrast, homozygous individuals had white spots throughout the body and less-pigmented eyes of normal size (Figure 3D). The RT-PCR analysis of the *Mitf^em1Cu/em1Cu^* mice tissues clearly detected a *Mitf* mRNA fragment shorter than that in the WT and indicated that the *Mitf^em1Cu^* mutation does not alter the open reading frame but yielded an in-frame deletion (Figure 3E). Sequence analysis of the RT-PCR products identified an in-frame deletion of 27 bp, which corresponded to *Mitf* amino acid residues 266–274 (Figure 3F). The *Mitf^em1Cu^* mutation engineered into the B6.C3H-*In(6)1J* inversion chromosome acted as a coat color marker and enabled the inversion to be easily tracked.

### Generation of a lethal allele on a chromosome balanced with B6.C3H*-In(6)1J* using the *i*-GONAD method

**Figure 4:**
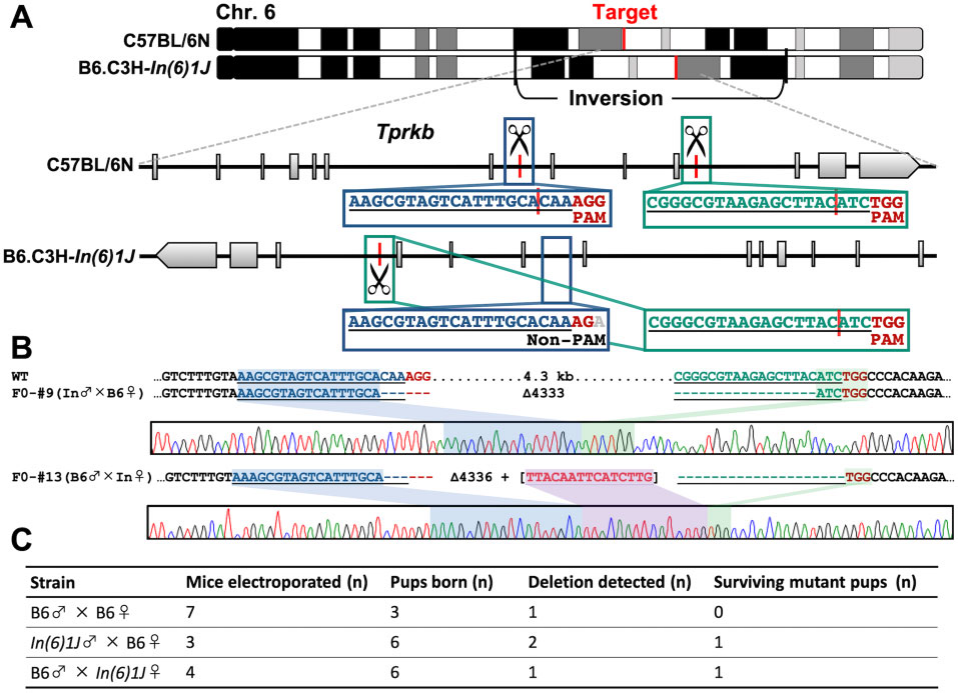
*Tprkb* deletion in mouse zygotes via targeting of the selected allele in F1 hybrid mice using the *i*-GONAD method. (A) Schematic representation of *Tprkb* deletion in F1 hybrid mice generated in the C57BL/6N allele only. (B) Alignment of sequences corresponding to *Tprkb* introns 7 and 10 genomic breakpoint junctions. (C) Summary of the experimental efficiency of *Tprkb* deletion via the *i*-GONAD method. B6, C57BL/6N.

To produce F0 mice carrying the embryonic lethal mutation, we designed a method wherein one of two alleles of the gene was selectively edited by *i*-GONAD-mediated mutagenesis in the presence of a non-targeted B6.C3H*-In(6)1J* in heterozygotes. We attempted to disrupt a potentially essential gene, *Tprkb* (encoding the *Tp53rk* binding protein), which was expected to result in lethal phenotypes based on a previous study (Braun *et al.* 2017); however, this has not been accurately determined. To induce a large deletion and complete knockout of *Tprkb*, we cut two sites using two gRNAs that selectively target one of the C57BL/6N (B6) alleles (Figure 4A). We electroporated the genome editing CRISPR/Cas9 mixture into the oviducts of three B6 females that mated with B6.C3H*-In(6)1J Mitf^em1Cu^* males, and seven B6 females that mated with B6 males were used as controls. Control B6/B6 strains had only three pups born through cesarean section, suggesting that most embryos died owing to the deletion of both *Tprkb* gene alleles. One of the three pups had a deletion mutation, but we could not obtain a surviving founder F0 (Figure 4C). In contrast, in B6/B6.C3H-*In(6)1J Mitf^em1Cu^* hybrid strains, we obtained six F0 pups via cesarean section and found that two had large deletions in the target locus, resulting in one viable F0 mouse (Figure 4, B and C). Similarly, B6.C3H-*In(6)1J* females that mated with B6 males successfully obtained a viable F0 mouse with *Tprkb* deletion (Figure 4, B and C). This approach used fewer animals than are required by conventional methods, which is beneficial in terms of animal welfare. Unlike in our previous study performed using F1 hybrid mice (Iwata *et al.* 2019), the new method avoids the need for a few generations of backcrossing to achieve genetic homogeneity. Thus, the strategy in which one allele in an essential gene is selectively targeted by *i*-GONAD-mediated gene editing enables researchers to efficiently generate a strain carrying the lethal allele.

The *i*-GONAD method reported by Ohtsuka *et al.* (2018) was confirmed to have comparable efficiency to microinjection. Hence, our method in this study would be as effective as the standard pronuclear injection methods.

### Confirmation of B6.C3H-*In(6)1J* inversion to balance a lethal mutation

**Figure 5:**
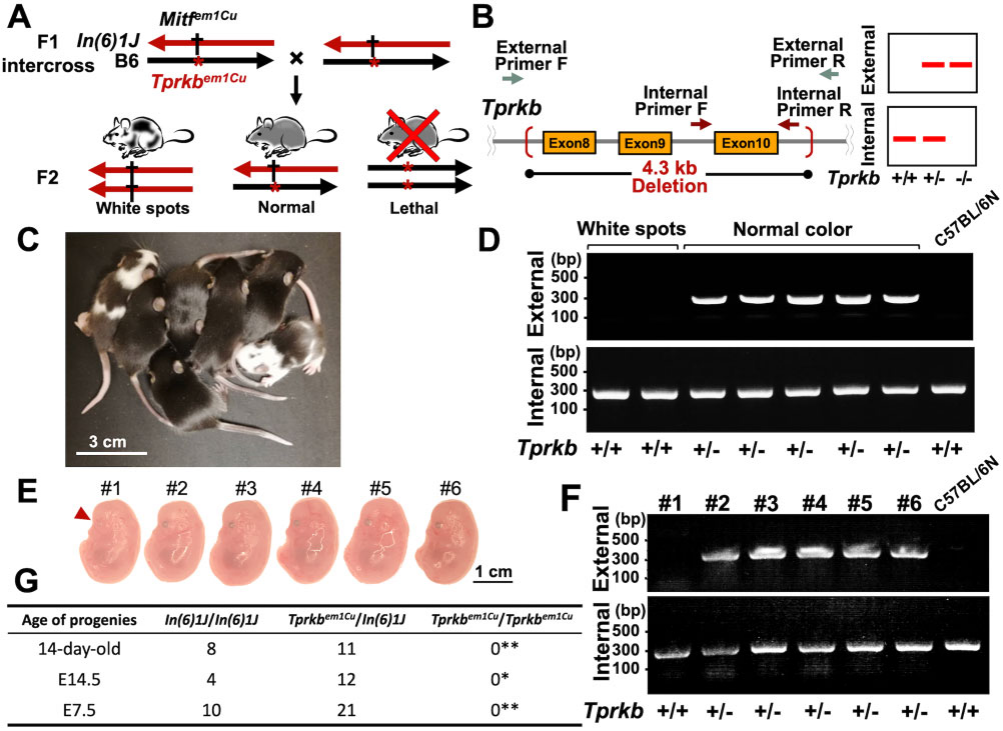
Chromosomal inversion B6.C3H*-In(6)1J* can balance a lethal mutation. (A) Overview of animal crossing schemes used to test whether B6.C3H-*In(6)1J* could balance a *Tprkb* mutation. Sibling matings between *Tprkb^em1Cu^*/*In(6)1J* mice generate three classes of F2 mice, *In(6)1J*/*In(6)1J*, *Tprkb^em1Cu^*/*In(6)1J*, and *Tprkb^em1Cu^*/*Tprkb^em1Cu^*, which can be distinguished by the presence of the *Mitf* mutation on the balancer chromosome. (B) Schematic representation of the PCR primer positions for detecting the *Tprkb* genotype. The external primers are designed to amplify only the *Tprkb^em1Cu^* product. The internal primers are only able to bind the wild-type product. The expected results of the PCR amplifications are shown for the three potential genotypes at the right of the figure. (C) Appearance of *In(6)1J*/*In(6)1J* and *Tprkb^em1Cu^*/*In(6)1J* pups at 14 days of age. *In(6)1J*/*In(6)1J* mice had a white spot phenotype. *Tprkb^em1Cu^*/*In(6)1J* mice were phenotypically normal. (D) Genotyping of seven pups was performed to confirm *Tprkb* genotypes. (E) Appearance of *In(6)1J*/*In(6)1J* and *Tprkb^em1Cu^*/*In(6)1J* fetuses at E14.5. The *In(6)1J*/*In(6)1J* fetus had less-pigmented eyes, indicated by the red arrowhead. (F) Genotyping of six fetuses was performed to confirm *Tprkb* genotypes. (G) Summary of *In(6)1J*/*In(6)1J*, *Tprkb^em1Cu^*/*In(6)1J*, and *Tprkb^em1Cu^*/*Tprkb^em1Cu^* offspring proportions. Asterisks indicate a significant difference, as determined using the chi-square test (*P < 0.05, **P < 0.01).

We examined whether B6.C3H-*In(6)1J* could balance a recessive lethal mutation within the inversion interval, as described in Figure 5A. A homozygous B6.C3H-*In(6)1J Mitf^em1Cu^* male was mated with a heterozygous female carrying a *Tprkb^em1Cu^* mutation, and the F1 trans-heterozygotes were further intercrossed. As mentioned previously, B6.C3H-*In(6)1J Mitf^em1Cu^* contains a coat color marker, which allows animal carriers to be easily identified. Following inbreeding of F1 mice, the balanced strain *Tprkb^em1Cu^*/B6.C3H-*In(6)1J Mitf^em1Cu^* segregated into two phenotypes: black progeny inherited the heterozygous *Tprkb^em1Cu^* mutation, whereas white spot progeny were genotyped as wild-type (Figure 5, B–D). We also confirmed all breakpoints of the inversion *In(6)1J* (Supplementary Figure S1). As homozygous *Tprkb^em1Cu^* mutants were not observed in live-born progeny, we collected embryos at embryonic days 14.5 (E14.5) and E7.5 and analyzed them. However, no *Tprkb^em1Cu^* homozygous embryo was found (Figure 5, E–G). These non-Mendelian ratios suggest that the *Tprkb* null mutation results in the death of homozygotes at the developmental stage earlier than E7.5 (Figure 5G). Previous studies showed that F0 embryos with CRISPR/Cas9 knockout of *Tprkb* exhibited primary microcephaly (Braun *et al.* 2017); however, most embryos injected with gRNA and Cas9 mRNA are genetically mosaic (Yen *et al.* 2014; Oliver *et al.* 2015). Thus, these phenotypes may be attributed to a combination of mutations. Therefore, B6.C3H-*In(6)1J* makes it easier for researchers to maintain lethal mutations and more efficient experiments in which lethal mutants can be analyzed.

## Conclusions

The B6.C3H-*In(6)1J* strains generated in this study will be deposited as frozen sperm at the RIKEN BioResource Research Center and Center for Animal Resources. Table 1 lists the stock balancer strains currently available via the public bioresource community. These balancers are genetically identical to the inbred strain, except for the inverted region and its surrounding region.

**Table 1:** Inversion balancer strains currently available via public bioresources.

Strains (Repository/Stock#)	Chr.	Covering	Genetic background		Phenotypes		References
			Inverted region	External region	In/+	In/In	
*In(D4Mit117; D4Mit281)1Brd* (MMRRC/031767-UCD)	4	96742762–130172215 bp	129S7	FVB	Light brown coat	Dark brown coat	Nishijima *et al.* 2003
*In(D4Mit281; D4Mit51)2Brd* (MMRRC/031768-UCD)	4	130172113–155046016 bp	129S7	FVB	Light brown coat	Dark brown coat	Nishijima *et al.* 2003
*In(4)56Rk* (JAX/001379)	4	4A1–4E2	DBA/2J	C57BL/6J	Retinal degeneration	Embryonic lethal	Roderick *et al.* 1997
*In(6)1J Mitf^em1Cu^*	6	63000846–120827193 bp	C3H/HeJ	C57BL/6N	Normal	White spots	Current study
*In(11Trp53; 11Wnt3)8Brd* (MMRRC/000055-UNC)	11	69580359–103817957 bp	129S7	C57BL/6J	Light ears and tail	Embryonic lethal	Zheng *et al.* 1999
*In(15)21Rk/J* (JAX/000920)	15	15A1–15E	DBA/2J	C57BL/6J	Normal	Embryonic lethal	Roderick 1983

In this study, the allele-specific *i*-GONAD method in B6.C3H-*In(6)1J* mice allowed us to efficiently generate recessive lethal strains without *ex vivo* handling of embryos. The edited lethal genes were stably maintained in heterozygotes, as recombination did not occur within this inversion interval. Such heritable lethal mutations are common in many human inherited disorders (Hrabe de Angelis *et al.* 2015; Dickinson *et al.* 2016; Meehan *et al.* 2017), and our method using mice with B6.C3H-*In(6)1J* will be a useful tool for disrupting and analyzing disease-causing essential genes. In addition, B6.C3H-*In(6)1J* are applicable in *N*-ethyl-*N*-nitrosourea mutagenesis screens (Supplementary Figure S2), as the balancers shown in Table 1 were previously used (Kile *et al.* 2003; Boles *et al.* 2009). Our strategy provides an easier method by which researchers can create lethal mutations and analyze the mechanisms of action of genes.
